# Diagnostic and Prognostic Potential of AKR1B10 in Human Hepatocellular Carcinoma

**DOI:** 10.3390/cancers11040486

**Published:** 2019-04-05

**Authors:** Johanna K. DiStefano, Bethany Davis

**Affiliations:** Diabetes and Fibrotic Disease Unit, Translational Genomics Research Institute, 445 N 5th Street, Phoenix, AZ 85004, USA; bdavis@tgen.org

**Keywords:** AKR1B10, liver cancer, hepatocellular carcinoma, chemoresistance, biomarkers

## Abstract

Hepatocellular carcinoma (HCC) is a leading cause of cancer-related death worldwide. Although diagnostic measures and surgical interventions have improved in recent years, the five-year survival rate for patients with advanced HCC remains bleak—a reality that is largely attributable to an absence of early stage symptoms, lack of adequate diagnostic and prognostic biomarkers, and the common occurrence of acquired resistance to chemotherapeutic agents during HCC treatment. A limited understanding of the molecular mechanisms underlying HCC pathogenesis also presents a challenge for the development of specific and efficacious pharmacological strategies to treat, halt, or prevent progression to advanced stages. Over the past decade, aldo-keto reductase family 1 member 10 (AKR1B10) has emerged as a potential biomarker for the diagnosis and prognosis of HCC, and experimental studies have demonstrated roles for this enzyme in biological pathways underlying the development and progression of HCC and acquired resistance to chemotherapeutic agents used in the treatment of HCC. Here we provide an overview of studies supporting the diagnostic and prognostic utility of AKR1B10, summarize the experimental evidence linking AKR1B10 with HCC and the induction of chemoresistance, and discuss the clinical value of AKR1B10 as a potential target for HCC-directed drug development. We conclude that AKR1B10-based therapies in the clinical management of specific HCC subtypes warrant further investigation.

## 1. Introduction

Hepatocellular carcinoma (HCC) is the most common form of liver cancer [1]. In the United States, the disease has an annual incidence of at least 6 per 100,000 individuals [2] and represents the fastest rising cause of cancer-related death [3]. A strong link exists between liver cirrhosis and HCC: cirrhosis is an important promoter of HCC formation and HCC is the most common cause of mortality in cirrhotic patients [4]. Conditions that promote cirrhosis, including viral infection, nonalcoholic fatty liver disease (NAFLD), alcohol overconsumption, aflatoxin, and genetic factors, are recognized risk factors for HCC development [3]. Given the rising rate of NAFLD worldwide, the incidence of HCC due to this etiology is also expected to increase [5].

While improvements in diagnostic methods and surgical techniques have resulted in better clinical management of HCC, the five-year survival rate (<15%) for advanced HCC is dismal [6]. This is due to a combination of factors, including heterogeneity of HCC, which presents with tumors spanning an array of different clinical and biological behaviors, diagnostic challenges [7], and the development of anti-HCC drug resistance [8]. The lack of markers that are specific for tumor type or disease stage represents a critical gap in the current understanding and treatment of HCC. In addition, the molecular mechanisms underlying the malignant transformation of hepatocytes are poorly understood [9] which further complicates the effective treatment of HCC.

Aldo-keto reductases (AKRs) are a group of cytosolic, monomeric proteins that catalyze simple cofactor-dependent oxidation-reduction reactions [10]. Together, this group of more than 190 enzymes clusters into 16 individual families based on similar sequence identity and function [11]. Among the AKR families, the well-characterized AKR1B subgroup has been associated with different human diseases, such as diabetic complications and specific cancers. In humans, the AKR1B subgroup encompasses AKR1B1, AKR1B10, and AKR1B15 [11]. Over the past decade, a number of experimental and clinical studies have emerged in the literature implicating AKR1B10 in the development and progression of HCC and the development of acquired resistance to HCC chemotherapeutic agents, and supporting a role for this enzyme as a biomarker for HCC diagnosis and prognosis. The purpose of this review is to summarize the evidence supporting a functional role for AKR1B10 in the development and progression of human HCC and evaluate the potential clinical value of AKR1B10 to serve as a biomarker for the diagnosis, prognosis, and clinical management of this disease.

### Discovery and Characterization of AKR1B10

AKR1B10 was identified and characterized by a trio of independent research groups in 1998. The first study used differential-display polymerase chain reaction to identify genes upregulated in HCC relative to surrounding non-tumor tissue [12]. Out of 67 amplified cDNA fragments that were highly or uniquely expressed in tumors, five were dominantly upregulated. Of these, only one showed expression in all tested HCC samples. High amino acid sequence homology was noted between this cDNA fragment as well as mouse fibroblast growth factor-induced gene (80%), mouse vas deferens protein (76%), and human aldose reductase (62%). Because of its sequence similarity with other members of the AKR superfamily, the authors named this gene hepatoma-specific aldose-reductase-related protein (HARP). In the second investigation of aldose reductase (AR) in human liver cancer, Cao et al. [13] identified a novel RNA band larger than the expected AR band in a human HCC sample. Determination of the cloned sequence revealed high homology with the corresponding region of AR, prompting the researchers to name the new entity, AR-like 1 gene (ARL-1). In that study, ARL-1 was abundantly expressed in >50% of HCCs examined. Sequence characterization revealed a transcript of 1337 base pairs, an ATG codon at nucleotide 70, and an open reading frame coding for a protein of 316 amino acids. The protein sequence determined from the primary cDNA sequence indicated >70% homology with AKR1B1. In a survey of different tissues, ARL-1 was most abundantly expressed in the small intestine and colon, with lower levels in the liver, thymus, prostate, testis, and skeletal muscle. ARL-1 was found to have a high activity to reduce aromatic aldehydes (i.e., nitrobenzaldehydes and to a lesser extent, 4-carbozybenzaldehyde), pyridine aldehydes, and aliphatic aldehydes (i.e., methylglyoxal and diacetyl). With the exception of certain hexoses, ARL-1 showed consistently higher activity compared to AKR1B1 for the majority of substrates assayed. In the third report, a clone was isolated from a screening of total RNA obtained from human small intestine using primers specific to Chinese hamster ovary (CHO) reductase [14]. Based on its sequence similarity to CHO reductase, the clone was determined to be a new member of the AKR superfamily. In that study, the highest expression of the gene was found in the adrenal gland and intestinal tract, with low levels observed in the liver.

AKR1B10 catalyzes the reduction of aliphatic and aromatic aldehydes, including the conversion of retinals to retinols, resulting in the depletion of retinoic acid, an important signaling molecule in cell proliferation and differentiation [15]. AKR1B10 also regulates fatty acid biosynthesis and lipogenesis [16], detoxifies cytotoxic carbonyls [17], and confers resistance to a number of chemotherapeutic agents used in the treatment of HCC, including anthracyclines and cisplatin (discussed below).

## 2. AKR1B10 and HCC

### 2.1. Association of Altered AKR1B10 Expression with HCC

The first study to investigate associations among HCC cells, histological grade, and levels of alpha-fetoprotein (AFP: an established tumor marker in HCC) applied two-dimensional difference gel electrophoresis in conjunction with mass spectrometry to profile proteomic content of tumor and adjacent non-tumor liver samples from 18 HCC patients [18]. AKR1B10 was overexpressed in HCC compared with non-tumor tissue and associated with histological grade, showing much higher levels in well-differentiated HCC compared to moderately differentiated tumor. An independent study reported increased AKR1B10 protein levels in well and moderately differentiated HCC compared to adjacent non-neoplastic tissue, however decreased levels in advanced tumor stages with a low grade of differentiation and an inverse correlation between AKR1B10 immunohistological staining and tumor proliferation [19]. While no significant relationships were observed between AKR1B10 immunostaining and vascular invasion, lymph node metastases, cirrhosis, and age, strong AKR1B10 staining was associated with longer survival time for HCC patients (315 versus 245 days for high versus low staining, respectively). AKR1B10 gene expression was significantly higher in HCC with liver cirrhosis (*n* = 22) and tumor-free cirrhotic liver samples (*n* = 22) [19]. This study was the first to suggest that AKR1B10 might be a useful marker for early primary liver tumors with well or moderate differentiation. Large-scale proteomic analysis confirmed dysregulated AKR1B10 expression in HCC liver tissues compared to normal liver [20].

A multicenter study recently validated AKR1B10 as a marker for the detection of HCC [21]. AKR1B10 levels were >18 times higher in serum samples from HCC patients compared to healthy individuals (1567.3 ± 292.6 pg/mL versus 85.7 ± 10.9 pg/mL). An optimal diagnostic cutoff of 267.9 pg/mL was determined using a training cohort of 519 participants. Serum AKR1B10 showed better diagnostic parameters of the area under the curve (AUC) 0.896, sensitivity 72.7%, and specificity 95.7%, than AFP (AUC 0.816, sensitivity 65.1%, and specificity 88.9%), although the combination of the two markers showed better diagnostic accuracy than either one alone. AKR1B10 also showed diagnostic potential for patients with early stage HCC and AFP-negative HCC. Similar findings were recently reported in a study involving 78 HCC and 63 non-HCC patients [22]. In an analysis of serum AKR1B10 levels and HCC incidence relative to fibrosis stage, severe fibrosis was found to be associated with high AKR1B10 levels in HCC and non-HCC patients [23].

A number of subsequent studies reported an association with clinicopathologic parameters of HCC. A retrospective analysis of liver samples from 168 HCC patients found significant associations between AKR1B10 expression and lower primary tumor classification (stronger AKR1B10 staining in pT1/pT2 versus pT3/pT4), presence of underlying viral hepatitis infection, and presence of cirrhosis, however not gender, histological grade, lymph or blood vessel infiltration, lymph node metastasis, local recurrence, or resection status [24]. HCCs classified as AKR1B10-positive showed lower values of proliferative cells and a smaller percentage of tumor cells expressing the proliferative-associated antigen Ki-67 compared with AKR1B10-negative cancers. A recent study confirmed increased AKR1B10 expression in early-stage HCC and showed higher AKR1B10 expression in moderately differentiated HCC compared with well-differentiated HCC, poorly differentiated HCC, and liver cirrhosis [25]. Immunohistochemical analysis of liver tissue discovered high AKR1B10 expression in 97% of HCCs examined, with little to no expression in adjacent hepatic tissue, hepatic adenomas, or focal nodular hyperplasia, demonstrating the potential use of this protein to distinguish HCC from benign liver lesions [26]. In that work, the authors also showed that AKR1B10 knockdown in vitro corresponded with increased apoptotic cell death, decreased colony formation and size, and improved response to doxorubicin. In an analysis involving children and older adults (≥80 years), AKR1B10 protein levels differed between HCC tissues and near-tumor tissues for HCC patients [27]. Interestingly, this study also revealed a significant difference in AKR1B10 expression according to age, with increased expression in older patients compared to levels in patients younger than 40 years. Results from the studies presented here are shown in Table 1.

Taken together, the evidence for the involvement of AKR1B10 in HCC is complicated and seemingly conflicting. Indeed, the paradoxical role of AKR1B10 in HCC tumor prognosis is well recognized. For example, while AKR1B10 promotes cell proliferation, AKR1B10 expression is nearly undetectable in HCC cells with high invasive capacity [28]. Likewise, although AKR1B10 overexpression is associated with reduced cell migration and invasion of highly invasive cells, silencing of AKR1B10 induced expression of vimentin and snail, which are known markers of epithelial mesenchymal transition or EMT [28]. AKR1B10 induction by specific factors was postulated to promote HCC cell proliferation through the depletion of retinoic acid in the early stages of primary HCC, however in advanced HCC, this pathway becomes inactivated, resulting in the downregulation of AKR1B10 (by other as-yet unknown factors) and leading to EMT and tumor metastasis.

### 2.2. AKR1B10 as A Prognostic Biomarker in HCC

A number of studies has explored the potential use of AKR1B10 levels in the prognosis of HCC. In HCC patients treated with surgical resection (*n* = 92) or liver transplantation (*n* = 76), AKR1B10 overexpression was significantly associated with lower tumor classification, underlying viral hepatitis, or cirrhosis [24]. In the resection group, a poorer prognosis was observed in patients with AKR1B10-negative HCCs compared to those with HCCs showing strong AKR1B10 staining. In HCC patients who underwent curative hepatectomy, high AKR1B10 expression was detected in nearly half of all 255 individuals and was associated with absences of major portal vein invasion and intrahepatic metastases, lower tumor stages, reduced alpha-fetoprotein (AFP) levels, and lack of early recurrence [32]. The major finding of this work demonstrated that high AKR1B10 expression was associated with longer recurrence-free survival and longer disease-specific survival (defined as the interval between date of surgery and date of HCC-related death) compared to low AKR1B10 expression. In HCC patients with high levels of the 14-3-3ε a protein associated with poor survival rates and higher incidence of metastasis, elevated AKR1B10 expression corresponded with better overall survival and disease-free survival and lower metastatic incidence compared with low expression [28]. In contrast, an unrelated study identified patients with positive 14-3-3ε staining and no increase in AKR1B10 (and decreased levels of a third protein, metallothionein-1) in primary tumors as having the worst overall and disease-free survival rates, as well as the highest risk for metastasis [33]. Additional reports of associations between favorable HCC prognosis following surgical resection and higher AKR1B10 expression are consistent with a dynamic role for this enzyme at different stages of HCC development and progression [30,31].

AKR1B10 expression is also associated with viral hepatitis and HCC risk. Hepatic gene expression profiling in 48 patients with chronic hepatitis C virus (HCV) infection identified 627 differentially expressed genes between HCV patients with normal or elevated levels of serum AFP [34]. Among these, AKR1B10 showed the most abundant change (26-fold). In normal liver tissue, AKR1B10 immunoreactivity was mainly observed in bile duct cells and rarely in the cytoplasm of hepatocytes, while in HCV samples, AKR1B10 immunoreactivity was pronounced in the cytoplasm of hepatocytes, and these AKR1B10-positive hepatocytes were mostly localized to the periportal zone. AKR1B10-positive areas were significantly greater in patients with higher AFP levels compared to control individuals and HCV patients with normal AFP levels. One of the most interesting aspects of this study was a matched case-control analysis to evaluate the risk of AKR1B10 expression for HCC development. During a minimum follow-up duration period of one-year post-biopsy, 20 HCV patients were diagnosed with HCC, and AKR1B10 expression was compared with HCV patients who did not develop HCC, matched by age, gender, and histological fibrosis stage. Not only was higher AKR1B10 expression observed in HCC cases, but AKR1B10 upregulation was also an independent risk factor for HCC development. A subsequent study by this team reported that the five-year cumulative incidence of HCC was ~23% and 2% in HCV patients with high and low AKR1B10 expression, respectively [35]. In subgroup risk analyses, hazard ratios attributed to elevated AKR1B10 expression (≥6%) were significant even in HCV patients considered to be at lower risk of HCC development, including individuals with a younger age, mild (versus severe) hepatic fibrosis, or those who experienced a sustained virological response through interferon-based antiviral treatment. In an unrelated study of HCV patients who had achieved sustained virological response, high AKR1B10 expression was an independent risk factor for HCC development and the five-year cumulative incidences of HCC development were ~14% and 0.5% in patients with high and low AKR1B10 expression, respectively [36]. Metabolomic profiles (high polyol levels) associated with HCV were retained in formerly HCV-infected individuals, suggesting that the effects of HCV on AKR1B10 expression might be long-lived [37]. In hepatitis B virus (HBV)-infected patients, AKR1B10 expression was recently associated with increased HCC risk at levels comparable to those in HCV patients [38].

At first glance, it would appear that the evidence for AKR1B10 as a biomarker for HCC is conflicting (Table 2). However, these results are consistent with results from experimental studies showing different contributions of AKR1B10 depending on HCC stage of development. For example, as noted earlier, Liu et al. [28] postulated that AKR1B10 plays dual roles depending on HCC stage: in early HCC, 14-3-3ε upregulates AKR1B10, resulting in decreased levels of retinoic acid and subsequent cell proliferation. In advanced HCC, the relationship between 14-3-3ε and AKR1B10 is repressed by as-yet unknown factors, leading to decreased levels of AKR1B10, which are associated with increased levels of EMT and metastasis. Although the mechanisms underlying these pathways require further investigation, they support a role for AKR1B10 as a marker of early HCC [18,24,32]. Based on the findings reported to date, we speculate that AKR1B10-positive cells may interact with other factors, such as HCV/HBV viral load or presence of cirrhosis to activate or repress additional pathways, thereby contributing to a number of specific HCC subtypes with variable prognoses. Focused investigations involving refined stratification of HCCs are necessary to address this hypothesis.

### 2.3. Molecular Mechanisms by Which AKR1B10 Contributes to The Development of HCC

Lipogenesis plays an important role in carcinogenesis [39] and a number of studies have provided evidence showing that AKR1B10 may contribute to the development and progression of cancer through its relationship with de novo fatty acid synthesis. In breast cancer cells, AKR1B10 was associated with acetyl-CoA carboxylase alpha (ACCA), the rate-limiting enzyme of de novo long chain fatty acid synthesis, and knockdown of AKR1B10 in cancer cells led to a significant reduction in both ACCA protein levels and fatty acid synthesis [16]. In the presence of a proteasome inhibitor, ACCA protein reduction was blocked, indicating that AKR1B10 prevents ACCA degradation through the ubiquitination-proteasome pathway [16]. These results suggested that by regulating the stability of ACCA, AKR1B10 affects fatty acid synthesis. A subsequent study by this team showed that AKR1B10 silencing resulted in decreased ACCA levels in colon (HCT-8) and lung cancer (NCI-H460) cells, leading to apoptotic cell death [40]. AKR1B10 knockdown also corresponded with a significant reduction (i.e., >50%) in levels of phospholipids, triglycerides, and free fatty acids, but not cholesterol, and a 2–3-fold increase in intracellular lipid peroxides [40]. Silencing of AKR1B10 in these cells also resulted in increased production of reactive oxygen species and decreased levels of mitochondrial cytochrome c levels; released cytochrome c was shown to activate caspase -3, leading to apoptosis. Combined, these results suggest that AKR1B10 knockdown might promote apoptosis by two mechanisms: one involving mitochondrial membrane impairments and the other through increased levels of cellular carbonyls. The authors hypothesized that AKR1B10 knockdown creates a vicious cycle involving increased cellular carbonyls, oxidative stress, and mitochondrial membrane lesions, leading to major effects on cell survival.

In HCC cells (Hep3B), knockdown of AKR1B10 expression resulted in cell cycle arrest and impairment of cell proliferation, indicating a tumorigenic effect that occurs through enhanced cell growth [41]. Integrative miRNA-mRNA analysis of RNA-sequencing and miRNA-sequencing data predicted that levels of miR-383-5p, a known tumor suppressor [42], were negatively associated with AKR1B10 expression, a finding concordant with results from experiments showing direct binding between the miRNA and the AKR1B10 3′ untranslated region [41]. Another mechanistic study identified AKR1B10 as the only gene showing strong correlation with interleukin-1 receptor-associated kinase 1 (IRAK1) expression in a HCC cell line [43]. Knockdown of AKR1B10 in Huh7 cells abrogated IRAK1-mediated sphere-forming ability, resistance to the tyrosine kinase inhibitor, sorafenib, and marker expression of tumor-initiating cells compared to normal Huh7 cells. A putative activator protein 1 (AP-1)-binding site was identified proximal to the AKR1B10 transcription start site, suggesting that AKR1B10 gene expression could be regulated by AP-1. Interestingly, IRAK1 knockdown resulted in dose-dependent downregulation of AP-1 promoter activity; these results warrant further exploration of the relationships among AKR1B10, IRAK1, and AP-1 in the development of HCC.

Reduced phospholipid levels concomitant with tumor cell apoptosis resulting from AKR1B10 inhibition [40] indicate that the oncogenic function of AKR1B10 might be mediated by these molecules. To address this hypothesis, hepatomas (HepG2) and hepatocytes (QSG-7701) were either co-cultured or QSG-7702 cells were treated with conditioned media from HepG2 cells [44]. Co-culturing did not affect the HepG2 cell number, however it increased the proliferation of QSG-7701 cells; conditioned media from HepG2 cells produced the same effect. AKR1B10 activity was found to be higher in lysates and medium from HepG2 cells compared to QSG-7701 cells. While AKR1B10 knockdown in HepG2 cells reduced AKR1B10 secretion, blocking of secreted AKR1B10 with an antibody did not affect the increase in QSG-7701 cell proliferation produced by co-culture of the two cell types, indicating the presence of a downstream signaling molecule mediated by cellular AKR1B10 in hepatoma cells. The authors noted that levels of sphingosine-1 phosphate (S1P) were higher in media from HepG2 cells compared to QSG-7701 cells and that increased QSG-7701 proliferation could be impaired in the presence of S1P inhibition. AKR1B10 knockdown led to reduced S1P levels in HepG2 media and inhibited cell growth in HepG2 cells, however not QSG-7701 cells. This work suggests that the AKR1B10-S1P signaling pathway contributes to enhanced QSG-7701 proliferation induced by co-culture with HepG2 cells; however, the extent to which this pathway operates in vivo remains to be seen.

To date, studies on potential molecular mechanisms by which AKR1B10 contributes to the development of HCC have been limited. In addition to the studies here, Liu et al. [28] presented evidence suggesting that AKR1B10 may exert different effects depending on HCC stage. As noted, upregulation of AKR1B10 by 14-3-3ε in early HCC results in depletion of retinoic acid, leading to cell proliferation. In contrast, this relationship appears to be inhibited in advanced HCC, resulting in decreased AKR1B10 levels, which are associated with increased levels of EMT and metastasis.

## 3. Clinical Significance of AKR1B10 in the Treatment of HCC

### 3.1. AKR1B10 and Chemoresistance

AKR1B10 contributes to the development of anticancer drug resistance, which is not surprising given the role of this enzyme in detoxification. In an early report, AKR1B10 was shown to metabolize the side-chain C13-ketonic group of daunorubicin and idarubicin (anthracyclines used for the treatment of liver cancer), resulting in acquired resistance to these compounds [45]. AKR1B10 was the most highly upregulated aldo-keto reductase during induction of doxorubicin resistance in gastric cancer cells, and inhibition of AKR1B10 decreased the migrating and invasive properties of these cells [46]. AKR1B10 also plays a role in the development of resistance to cisplatin (CDDP), a chemotherapeutic increasingly used in the treatment of HCC [47]. As with doxorubicin resistance, AKR1B10 expression was significantly upregulated during acquisition of CDDP resistance and CDDP cytotoxity increased in parallel with AKR1B10 knockdown. Overexpression of AKR1B10 suppressed accumulation and cytotoxicity of 4-hydroxy-2-nonenal, an α, β-unsaturated hydroxyalkenal produced during lipid peroxidation by CDDP treatment, suggesting that AKR1B10 might induce CDDP resistance by inhibiting oxidative stress by the drug. Interestingly, CDDP resistance was associated with decreased expression of peroxisome proliferator-activated receptor-γ (PPARγ) in gastric adenocarcinoma (MKN45) and colon cancer (LoVo) cells, while conversely, PPARγ overexpression increased cell sensitivity to CDDP toxicity [47]. AKR1B10 overexpression corresponded with reduced PPARγ expression which was attenuated by co-treatment with oleanolic acid, an AKR1B10 inhibitor. Treatment of gastric cancer cells with both PPARγ ligand and oleanolic acid restored most of the CDDP cytotoxicity. Together, these results imply that AKR1B10 mediates CDDP resistance through a PPARγ-dependent mechanism and indicate that a pharmacological strategy involving an AKR1B10 inhibitor and PPARγ agonist may prevent the development of CDDP resistance in gastrointestinal cancer. While the results from this study and the doxorubicin study [46] implicate AKR1B10 in the induction of chemoresistance, these mechanisms need to be explored in HCC to establish whether similar events occur in liver cancer cells.

### 3.2. AKR1B10 Inhibitors

Given its role in cellular processes underlying the development of HCC and its ability to metabolize cancer drug therapies, AKR1B10 may represent a potential target for the treatment and prevention of liver cancer. Indeed, the development of specific inhibitors of AKR1B10, an endeavor complicated by the high similarity in primary and tertiary structure to AKR1B1, has been an active area of research for several years. In general, AKR1B10 inhibitors fall into four major categories, including known aldose reductase inhibitors, synthetic compounds, endogenous substances, and natural derivatives. Because the names, structures, and selectivity for AKR1B10 of these inhibitors have recently been reviewed in detail [48], we will focus only on inhibitors selective to AKR1B10 in this review. A list of representative AKR1B10 inhibitors and the concentrations required to achieve half maximal inhibition of AKR1B10 (IC50) are shown in Table 3 and are discussed briefly here. For example, the natural derivative bisdemethoxycurcumin (BDMC) has 85-fold selectivity for AKR1B10 over AKR1B1 with an IC_50_ 60 ± 9 nM BDMC [49], indicating that it has strong selectivity for AKR1B10, although at a lower level than other inhibitors shown in Table 3. Molecular docking and mutagenesis experiments determined that the selectivity of BDMC is dependent on interactions with the Gln114, Val301, and Gln303 residues in AKR1B10 [50]. Like BDMC, the caffeic acid phenethyl ester (CAPE) derivative, 10c, also interacts with the Gln114, Val301, and Gln303 residues in the AKR1B10 tertiary structure; however, the interaction between the 2-methoxy side chain of 10c with Val301 yields a greater specificity for AKR1B10 over AKR1B1 (IC_50_ 6.2 ± 0.01 nM and 790-fold selectivity for AKR1B10/AKR1B1) [51].

AKR1B10 and AKR1B1 exhibit differential substrate specificity and inhibitory effects of steroid hormones and bile acids and these differences have been exploited in the development of specific AKR1B10 inhibitors. For example, while isolithocholic acid, which binds to and inhibits AKR1B10 in a manner similar to tolrestat, an aldose reductase inhibitor, the interaction between the C-24 carboxylate of the molecule and Lys125 at the AKR1B10 binding site makes it a more potent and highly selective AKR1B10 inhibitor compared to that drug [52].

Another natural-based selective AKR1B10 inhibitor, oleanolic acid, was shown to form bonds with Gln303 and Val301 in the AKR1B10 binding site; these binding interactions were not present with AKR1B1, which likely accounts for this compound showing the highest selectivity for AKR1B10 (1378-fold selectivity) [54]. A polyhalogenated derivative, MK204, forms a strong halogen interaction between its aryl group and Trp112 of the AKR1B10 active site making it a decent AKR1B10 inhibitor, IC_50_ 80 ± 0.01 nM [43]. A novel polyhydroxy steroid, compound 6, has a 120-fold selectivity preference of AKR1B10 than AKR1B1 and IC_50_ of 830 nM making it the most effective AKR1B10 from this family, in part due to its strong interaction with the catalytic domain and interactions with Gln303 and Val301 [55].

In sum, there are a number of potent and selective AKR1B10 inhibitors currently available. An understanding of important features for consideration in the design of such compounds has been improved through analysis of these inhibitors: the inhibitor must be able to fit properly within the binding site of AKR1B10 and interactions with the Gln114, Val301, and Gln303 residues are critical. Efforts to design improved AKR1B10 inhibitors remains to be an ongoing area of research activity, while their effectiveness in the treatment of HCC remains to be seen.

## 4. Conclusions

Hepatocellular carcinoma (HCC) is the third leading cause of cancer-related death worldwide [56]. Poor outcomes for patients with advanced HCC [6] are largely due to the lack of biomarkers to identify tumors in the early stages prior to progression to advanced stages and metastasis, resistance to pharmacological interventions, and a high degree of intratumor heterogeneity [57]. The identification of biomarkers for the different pathophysiological stages of HCC are critical to improve early disease detection and enable early implementation of chemotherapy or surgical resection to prevent progression to deadlier advanced stages and tumor metastases. AKR1B10 is emerging as a promising biomarker for HCC: higher AKR1B10 expression is correlated with better long-term outcomes, such as increased survival rate and lower metastatic incidence [28]. AKR1B10 expression is also higher in more advanced states of HCC, indicating that it would serve as a useful biomarker for a good prognosis in patients with HCC [44].

AKR1B10 may likewise represent a potential therapeutic target for the treatment of HCC. A number of experimental studies have shown that silencing of AKR1B10 prevents tumor growth and metastasis and induces cell death. Further, inhibition of AKR1B10 in HCC will allow for a better response to chemoresistance because of its ability to metabolize a wide range of cancer drug therapies. Issues stemming from the high sequence similarity among members of the AKR family present certain obstacles to the development of specific AKR1B10-based treatments, although a number of inhibitors have already been designed with strong selectivity for AKR1B10. Results from several studies support the use of inhibitors as anti-proliferative agents against AKR1B10-overexpressing cancers like HCC and as adjuvant drugs for overcoming chemoresistance in HCC.

Finally, despite awareness of risk factors for the development of HCC and advances in the diagnosis and clinical management of the disease, the molecular mechanisms underlying hepatocarcinogenesis remain poorly understood. AKR1B10 may play a key role in HCC based on its biological properties and through a number of mechanisms including, however not limited to, lipogenesis, oxidative stress, detoxification of cytotoxic reactive carbonyls, and regulation of S1P. AKR1B10 expression levels during early stage HCC might also be exploited for early cancer detection in those patients with AKR1B10-expressed cells. Overall, much remains to be learned about the molecular role of AKR1B10 in HCC and the mechanisms by which AKR1B10 promotes disease onset and progression; however, the value of AKR1B10-based diagnostic methods and therapies in the clinical management of specific HCC subtypes warrant further investigation.

## Figures and Tables

**Table 1:** AKR1B10 expression patterns in Hepatocellular carcinoma (HCC).

Study Design	Assay	AKR1B10	Reference
18 HCC versus 18 non-HCC	protein	Increased	[18]
22 HCC versus 22 NT ^1^	mRNA	Increased	[19]
210 HCCs versus non-neoplastic liver tissue	protein	Increased	
89 HCC versus 33 BLL ^2^	protein	Increased	[26]
61 HCC versus 8 NL ^3^ and 61 NT	protein	Increased	[29]
158 HCC versus NT	mRNA	Increased	[30]
110 HCCs versus adjacent NT	mRNA	Increased	[31]
44 HCC versus 37 non-HCC	protein	Increased	[25]
280 HCC versus 168 NT	protein	Increased	[27]

^1^ NT: non-tumorous tissue; ^2^ BLL: benign liver lesions; ^3^ NL: normal liver.

**Table 2:** Diagnostic and prognostic impact of AKR1B10 levels in HCC.

Study Sample	Main Findings	Prognosis ^1^	Reference
168 HCCs with viral and non-viral etiology	AKR1B10 overexpression associated with lower pT-classification Loss of AKR1B10 correlated with increased proliferative activity Poorer prognosis in patients with AKR1B10-negative HCCs compared with patients with AKR1B10- positive HCCs	Favorable	[24]
48 HCC with hepatitis C virus (HCV)	≥6% up-regulation of AKR1B10 associated with ≥21-fold relative risk of HCC	Poor	[34]
255 HCCs with viral and non-viral etiology	High AKR1B10 expression independently predicted longer RFS ^2^ and longer disease-specific survival.	Favorable	[32]
109 HCCs with viral and non-viral etiology	AKR1B10 expression associated with free surgical margins, early BCLC ^3^ staging, and lack of metastasis Higher AKR1B10 expression associated with better OS ^4^, progression-free survival, and lower metastatic risk	Favorable	[28]
26 HCC with viral and non-viral etiology	Lower AKR1B10 expression was associated with worse RFS and OS.	Favorable	[30]
43 HCC with HCV	High AKR1B10 expression independently predicted HCC. 5-year cumulative incidences of HCC were 22.8% and 2.2% in patients with high and low AKR1B10 expression, respectively.	Poor	[35]
8 HCC with HCV	High AKR1B10 expression was the only independent risk factor for HCC. 5-year cumulative incidences of HCC were 13.7% and 0.5% in patients with high and low AKR1B10 expression, respectively.	Poor	[36]
13 HCC with HBV	High AKR1B10 expression independently predicted HCC. 5-year cumulative incidences of HCC were 20.6% and 2.6% in patients with high and low AKR1B10 expression, respectively.	Poor	[38]
110 HCC with HBV	Higher AKR1B10 expression associated with higher DFS ^5^ and OS and low risk of early HCC recurrence	Favorable	[31]

^1^ Based on high AKR1B10 levels; ^2^ RFS: recurrence-free survival; ^3^ BCLC: Barcelona Clinic Liver Cancer; ^4^ OS: overall survival; ^5^ DFS: disease-free survival.

**Table 3:** Representative AKR1B10 inhibitors.

Name	IC_50_^1^ AKR1B10	IC_50_ AKR1B1	Ratio ^2^	Reference
**BDMC ^3^**	60 nM	5100 nM	85	[49]
**10c ^4^**	6.2 nM	4900 nM	790	[51]
**Isolithocholic acid**	27 nM	6900 nM	256	[52]
**MK204 ^5^**	80 nM	21,700 nM	271	[53]
**Oleanolic acid**	90 nM	124,000 nM	1378	[54]
**Polyhydroxy steroid 6 ^6^**	830 nM	>100,000 nM	>120	[55]

^1^ IC_50_: half maximal inhibitory concentration; ^2^ Selectivity IC50 ratio of AKR1B1/AKR1B10; ^3^ BDMC: bisdemethoxycurcumin; ^4^ 10c: 3-(4-hydroxy-2-methoxyphenyl)acrylic acid 3-(3-hydroxyphenyl)propyl ester; ^5^ MK204: 2-(5-chloro-2-((2,3,4,5,6-pentabromophenyl)methylcarbamyl)phenoxy)acetic acid; ^6^ Polyhydroxy steroid 6: Androst-3β,5α,6β,19-tetrol.
